# Retraction Note: Transcription elongation factor A-like 7, regulated by miR-758-3p inhibits the progression of melanoma through decreasing the expression levels of c-Myc and AKT1

**DOI:** 10.1186/s12935-024-03472-5

**Published:** 2024-08-14

**Authors:** Xilin Liu, Xianji Song, Hong Li

**Affiliations:** 1 Department of Hand Surgery, China Japan Union Hospital of Jilin University, Changchun, 130033 Jilin China; 2 Orthopaedic Surgery, China Japan Union Hospital of Jilin University, Changchun, 130033 Jilin China; 3 Emergency Medical of China Japan Union Hospital of Jilin University, No. 126 Xian Tai Street, Changchun, 130033 Jilin China

**Retraction Note to:** Cancer Cell International (2021) 21:43

10.1186/s12935-020-01737-3

The Editors in Chief retracted this article because of significant concerns regarding a number of Figures presented in this work, which question the integrity of the data. The authors have contacted the journal requesting a retraction and stating some of the figures presented here did not match the original data. After publication, overlap was detected with figures in articles submitted and published within a close time frame including, but not limited to ([1–6], now retracted). Specifically, Figs. 2i, j, o and p, 4e, f, g and h and 6c. Furthemore, it appears there’s a small image overlap between Fig. 6e mimics + Lenti-NC 24 h and Fig. 6f mimics + Lenti-NC 24 h.

The Editors-in-Chief therefore no longer have confidence in the integrity of the data in this article.

All authors agree to this retraction.
